# Driving performance in the morning after bedtime vornorexant administration: A randomized clinical trial using a driving simulator

**DOI:** 10.1111/pcn.13888

**Published:** 2025-08-23

**Authors:** Yusuke Miyazaki, Kunihiro Iwamoto, Daiji Kambe, Yumiko Imadera, Isao Matsushita, Norio Ozaki

**Affiliations:** ^1^ Development Headquarters, Taisho Pharmaceutical Co., Ltd. Tokyo Japan; ^2^ Department of Psychiatry, Graduate School of Medicine Nagoya University Nagoya Japan; ^3^ Pathophysiology of Mental Disorders Nagoya University Graduate School of Medicine Nagoya Japan

**Keywords:** cognitive function, driving performance, driving simulator, insomnia, vornorexant

## Abstract

**Aim:**

Sedative hypnotics can impair driving performance to a degree comparable to blood alcohol concentrations of 0.05%–0.08%. This study aimed to evaluate the residual effect of vornorexant, a novel orexin receptor antagonist, on next morning driving performance relative to a predefined clinically meaningful threshold.

**Methods:**

Participants received 10 and 20 mg of vornorexant and placebo for eight consecutive nights and zopiclone on Days 1 and 8 in a four‐way crossover and double‐blind manner. Driving performance at 9‐h post‐dose was assessed measuring the standard deviation of the lateral position (SDLP) on Days 2 and 9 (days after single and repeated administration) using a validated driving simulator. Pharmacokinetics were also evaluated.

**Results:**

Sixty‐one participants were randomized and 55 completed the study. The mean of drug‐placebo changes in SDLP (ΔSDLP) following vornorexant 10 and 20 mg were 0.767 and 2.132 cm on Day 2, and −0.422 and −0.040 cm on Day 9, respectively, whereas approximately 6 cm in zopiclone. All the upper bounds of the 90% confidence intervals of these changes were below the predefined clinically significant threshold. Symmetry analysis found no differences in the proportion of participants with ΔSDLP exceeding the threshold in either impairing or improving from 0 cm, at vornorexant 10 mg on Days 2 and 9 and 20 mg on Day 9. Pharmacokinetic analysis showed consistent plasma concentrations of vornorexant in single and repeated dosing.

**Conclusions:**

Single and repeated administration of 10 and 20 mg vornorexant induced no clinically meaningful impairment in driving performance in healthy Japanese participants.

The use of hypnotics at bedtime to treat sleep disturbances raises concerns about “carry‐over” effects, where pharmacological activity persist into the next day beyond the intended period. The carry‐over effects of hypnotics cause depressant or sedative effects in the central nervous system (CNS), which can lead to various deleterious consequences for daily activities, including driving. Epidemiological studies have shown that the use of hypnotics increases the risk of traffic accidents^1^, ^2^, ^3^ and most benzodiazepine receptor agonists have presented a detrimental driving performance in clinical driving studies.^4^ A policy brief issued by the world health organization (WHO) also states that there is a growing concern in many countries about the risk of impaired driving performance led by CNS drugs, including benzodiazepines used as anxiolytics and sleep‐aid.^5^ Therefore, it is important to assess the impact of CNS drugs on driving performance.

In this context, the Food and Drug Administration (FDA) developed guidelines to evaluate drug effects on the ability to operate a motor vehicle and required pharmaceutical companies to adequately evaluate the effects on driving performance following CNS drug administration.^6^ These guidelines allow either an on‐the‐road test (ORT) or driving simulators (DS) to be used in driving studies. The Ministry of Health, Labour and Welfare of Japan also released a guideline for the assessment of driving performance and made a similar description.^7^, ^8^, ^9^ The ORT is validated and available only in the Netherlands; therefore, DS is the sole option in other countries. Iwamoto *et al*. established a new DS platform that showed high test–retest reliability, threshold calculations based on blood alcohol concentration, and assay sensitivity to well‐established positive control drug.^10^, ^11^ This is a novel option for the assessment of drug effects on driving performance.

Vornorexant (TS‐142), discovered by Taisho Pharmaceutical, is a novel and potent dual orexin receptor antagonist. Orexins are neuropeptides that regulate sleep/wake cycles *via* their corresponding receptors OX_1_ and OX_2_. Based on these physiological properties, vornorexant have been developed for insomnia treatment. Vornorexant has shown feasible pharmacokinetic profiles to achieve rapid onset of pharmacological action with a short half‐life.^12^ Moreover, pharmacodynamic assessment of vornorexant by several endpoints (Karolinska sleepiness scale [KSS], digit symbol substitution test [DSST], and psychomotor vigilance test [PVT] scores) revealed only subtle and inconsistent differences between vornorexant and placebo were observed at 9 h post‐dose, whereas more potent and clearer effects were observed at earlier time points (1 and 4 h post‐dose), highlighting the rapid elimination characteristic of vornorexant. Based on these results, vornorexant is expected to reduce the risk of next‐day residual effects. Importantly, a Phase 2a study demonstrated that vornorexant significantly improved objective measures of sleep in insomnia patients and was well tolerated.^13^ Vornorexant is under review for the treatment of insomnia by the Pharmaceuticals and Medical Devices Agency (PMDA) as of the time of submission. This study aimed to evaluate the residual effect of vornorexant on next morning driving performance following single and repeated doses of 10 mg (expected highest therapeutic strength) or 20 mg (extra‐therapeutic strength), compared to a clinically meaningful threshold, in healthy Japanese participants using validated and standardized DS. We hypothesized that vornorexant would result in significantly less impairment than clinically meaningful impairment, equivalent to a blood alcohol concentration (BAC) of 0.05%.

## Methods

### Participants

Healthy Japanese non‐elderly and elderly male and female volunteers were eligible if they had driven a car regularly for at least 3 years, had a regular sleep pattern, showed a physically and psychologically reliable DS operation, and had no visual disability.

Participants were excluded from the study if they failed the DS screening test (≥60 cm of standard deviation of lateral position [SDLP] or a course out from a traffic lane) and reported any of the following episodes within a specified duration prior to the study; a drug intake including over‐the‐counter medications within a week before screening, a hypnotic/sedative drug intake within 4 weeks before screening, traveling six time‐zones or more or any irregular shift work, and drinking alcohol before bedtime on a daily basis.

All participants provided written informed consent before enrollment, and their anonymity was maintained throughout the study.

### Design

This randomized, double‐blind, placebo‐ and zopiclone‐controlled, four‐way crossover study was conducted between January 16, 2021 and January 5, 2022 at Souseikai Fukuoka Mirai Hospital (Fukuoka, Japan). This study was approved by the Hakata Clinic Institutional Review Board and was conducted in compliance with the guidelines of the Declaration of Helsinki and Japanese Good Clinical Practice. This study was registered at ClinicalTrials.gov (NCT04696952) and jRCT (jRCT2071200075).

**Fig. 1:**
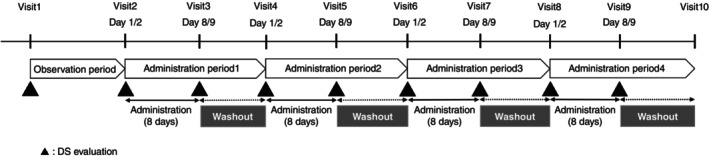
Study design. DS, driving simulator.

The present study consisted of a screening period with a first visit and four administration periods with eight visits during hospitalization, followed by a follow‐up visit (Fig. 1). Participants were randomized using a Williams design to one of four treatment sequences (vornorexant 10 mg [VOR10], vornorexant 20 mg [VOR20], zopiclone 7.5 mg [ZOP], and placebo [PBO]) in a 1:1:1:1 ratio at visit 2. Drug doses were chosen in accordance with the FDA^6^ and Japanese regulatory guidelines^7^, ^8^, ^9^; vornorexant 10 mg was considered the highest therapeutic dose and 20 mg as the extratherapeutic dose. To ensure comparability across conditions, ZOP was used as a positive control, as it has been reported to demonstrate assay sensitivity in both ORT and DS.^14^, ^15^, ^16^, ^17^ Unlike VOR10, VOR20, and PBO, which were administered nightly for 8 days, ZOP was administered only on Days 1 and 8 to avoid tolerance development, as it is known to rapidly induce receptor desensitization with repeated use^18^ and FDA determined this dosing schedule in the guidance.^6^ This design is consistent with previous trials that assessed the residual effects of hypnotic agents on driving performance.^14^, ^15^, ^16^, ^17^ Oral formulations of vornorexant and a matching placebo were prepared by the sponsor. ZOP was visually distinguishable from the other drugs, therefore, investigational drugs were administered in an opaque drug container to ensure blinded conditions.

The detailed schedule of all assessments for each administration period is shown in Table S1. Briefly, the participants were hospitalized on Days 1 and 8 and received the investigational drugs before bedtime. Participants performed DS tests and other assessments after waking on Days 2 and 9.

### Pharmacodynamics

Driving tasks were conducted 9 h after dosing on the morning of Days 2 and 9. DS testing was carried out as previously reported.^10^, ^11^ Participant manipulations of the steering wheel, brake pedal, and accelerator (Driving Force GT; Logicool) were recorded *via* a connected Windows PC, and simulated driving scenes were projected onto an 80‐in. screen using a liquid crystal projector (EB‐X05, Epson, Nagano, Japan). The simulated scenario was also equivalent to the previous scenario with a highway comprised of two lanes (dual‐carriageway) and gentle curves in each direction. The participants were instructed to continue driving at the center of the left side of the lanes while maintaining 100 km/h as much as possible for 60 min. Raw data were recorded at 20 ms intervals, and the following outcomes were computed: SDLP (primary outcome) was the standard deviation of the distance between the center line of the road and the right edge of the car, indicating swerving of the car; inappropriate line crossing (ILC), total course out (TCO), and standard deviation of speed (SDS) were evaluated as secondary outcomes. Driving tests were also performed on Days 1 and 8 to familiarize the participants with the DS.

Exploratory outcomes included a sleep questionnaire with self‐reported sleep parameters such as sleep latency (sSL), wake time after sleep onset (sWASO), and number of awakenings (sNAW) filled in by the participants immediately after waking in the morning. In addition, the Japanese versions of the KSS, DSST (correct answers), Word recall test, and POMS 2 total mood disturbance (TMD) were conducted according to previously published reports.^11^, ^19^, ^20^, ^21^, ^22^

### Safety assessments

Adverse events (AEs), adverse drug reactions, body weight, vital signs, standard 12‐lead electrocardiogram (ECG), laboratory tests, and the Columbia‐Suicide Severity Rating Scale (C‐SSRS) were evaluated as safety endpoints. AEs were tabulated by administration group and summarized according to the Japanese version of the Medical Dictionary for Regulatory Activities (MedDRA, Version 24.1).

### Pharmacokinetics

Blood samples were collected by venipuncture 0.5 h after driving (corresponding to 10.5 h after drug administration). Plasma vornorexant concentrations were determined using high‐performance liquid chromatography–tandem mass spectrometry (calibration range: 0.100–100 ng/mL, CMIC Pharma Science Corporation [Kobe, Japan]). Serum ZOP concentrations were measured using a high‐performance liquid chromatography‐tandem mass spectrometer (linear calibration range: 1.00–200 ng/mL, LSI Medience Corporation [Tokyo, Japan]).

### Sample size

The sample size was estimated based on two power calculations for the assay sensitivity to ZOP and the primary endpoint evaluation of vornorexant. The mean and standard deviation of ΔSDLP between ZOP and placebo were assumed to be 3.752 and 6.291 cm, respectively, based on a previous report.^11^ The necessary number of participants to obtain >90% statistical power was calculated as 49 for the lower bound of 90% confidence interval (CI) of ΔSDLP to exceed 0 cm. The mean and standard deviation of ΔSDLP between vornorexant and placebo were assumed to be 3.076 and 5.676 cm, respectively, based on previous reports.^10^, ^11^ The necessary number of participants was calculated as 16 for the upper bound of 90% CI of ΔSDLP to be below a clinically significant threshold of 9.229 cm with over 90% statistical power, the threshold determined as driving performance impairment in participants with blood alcohol concentrations of 0.05%.^11^ Based on these calculations and to account for potential dropouts, the sample size to ensure 90% statistical power for both ZOP and vornorexant assessment was determined to be 52 participants.

### Statistical analysis

The pharmacodynamic, safety, and pharmacokinetic analysis sets were defined as a population of participants who; had available pharmacodynamic outcomes without significant protocol violations; received at least one administration of investigational drugs; and had available pharmacokinetic outcomes with at least one administration of investigational drugs without pharmacokinetic‐related protocol violations.

The means of ΔSDLP were estimated by a model including administration group, sequence, administration period, timepoint, age category, sex, and the interaction between administration group and timepoint as fixed effects, and the participant as a random effect. Symmetry analysis for ΔSDLP were conducted using generalized sign tests to compare the proportion of participants with ΔSDLP ≥9.23 cm (reflecting impairment) and the frequency of participants with ΔSDLP of ≤ −9.23 cm (reflecting improvement) for each administration group at each timepoint. No missing data were imputed.

The clinically significant threshold was estimated for different numbers of decimal places (9.229 cm in mean analysis and 9.23 cm in symmetry analysis) for the following reason: individual SDLP was computed at two decimal places and mean SDLP was analyzed at three decimal places.

Multiple statistical tests for the primary endpoint were planned using a fixed‐sequence procedure (low‐dose to high‐dose) as following: given the upper bound of 90% CIs of ΔSDLP in VOR10 at both Day 2 and 9 fell below 9.229 cm (showing a lack of clinically meaningful impairment), the same analysis was subsequently performed in VOR20. For each of VOR10 and VOR20, given the upper bound of 90% CIs of ΔSDLP at both Days 2 and 9 were less than 9.229 cm, it was judged that no clinically meaningful impairment existed in each group. In this case, an overall alternative hypothesis is the intersection of an alternative hypothesis at each time point, and therefore multiplicity for time points is not necessary to be considered.

In the *post hoc* analysis, point estimates and 95% CI of the change in ΔSDLP from Day 2 to Day 9 in VOR10, VOR20 and ZOP were calculated based on the t‐distribution in each group.

Linear regression analysis was performed using vornorexant plasma concentration (ng/mL) as the independent variable and ΔSDLP (cm) as the dependent variable to evaluate the correlation between ΔSDLP and plasma concentration.

SAS (SAS Institute Inc., Japan; version 9.4) was used for all statistical analyses.

## Results

### Participant disposition and background

Sixty‐one participants were randomized, and of them six participants were discontinued from the study. The study flow diagram is shown in Fig. S1. One participant was excluded from the pharmacodynamic analysis because of a protocol deviation (noncompliance with contraception). A total of 60 participants were included in the pharmacodynamic analysis set (41 males, 19 females), with a mean (± standard deviation [SD]) age of 47.7 (±15.4) years (47 adults: defined as <65 years, 13 elderly: defined as ≥65 years), and mean weight, body mass index and annual driving experience of 62.53 (±7.55) kg, 22.23 (±1.61) kg/m^2^ and 8002.8 (±5833.9) km/year, respectively.

### Primary endpoint

The point estimates (90% CIs) in ΔSDLP on Days 2 and 9 were 0.767 (−0.633, 2.166) cm and −0.422 (−1.884, 1.040) cm in VOR10, and 2.132 (0.731, 3.533) cm and −0.040 (−1.498, 1.417) cm in VOR20, respectively. The upper bounds of the 90% CI for these ΔSDLP values were all below the 9.229 cm threshold, indicating that vornorexant did not produce any clinically meaningful impairment in next morning driving ability. The lower bound of the 90% CI on Day 2 in VOR20 exceeded 0 cm (Table 1).

**Table 1:** SDLP in all administration groups and changes from placebo in vornorexant 10 and 20 mg and zopiclone on Days 2 and 9.

		SDLP (cm)		ΔSDLP (cm)		
Group	Day	*n*	Mean ± SD	*n*	Point estimate	90% CI
PBO	2	55	35.751 ± 8.713	‐	‐	‐
	9	56	35.679 ± 8.710	‐	‐	‐
VOR 10 mg	2	56	36.569 ± 9.936	55	0.767	[−0.633, 2.166]
	9	56	35.234 ± 9.044	56	−0.422	[−1.884, 1.040]
VOR 20 mg	2	57	37.742 ± 9.341	54	2.132	[0.731, 3.533]
	9	57	35.796 ± 8.729	56	−0.040	[−1.498, 1.417]
ZOP	2	57	42.714 ± 10.948	53	6.174	[4.768, 7.579]
	9	55	41.031 ± 10.968	53	5.427	[3.951, 6.904]

CI, confidence interval; PBO, placebo; SD, standard deviation; SDLP, standard deviation of lateral position; VOR, vornorexant; ZOP, zopiclone.

In the ZOP treatment group, ΔSDLP on Days 2 and 9 were 6.174 (4.768, 7.579) cm and 5.427 (3.951, 6.904) cm, respectively. The lower bounds of the 90% CI exceeded 0 cm at these time points, confirming assay sensitivity.

**Fig. 2:**
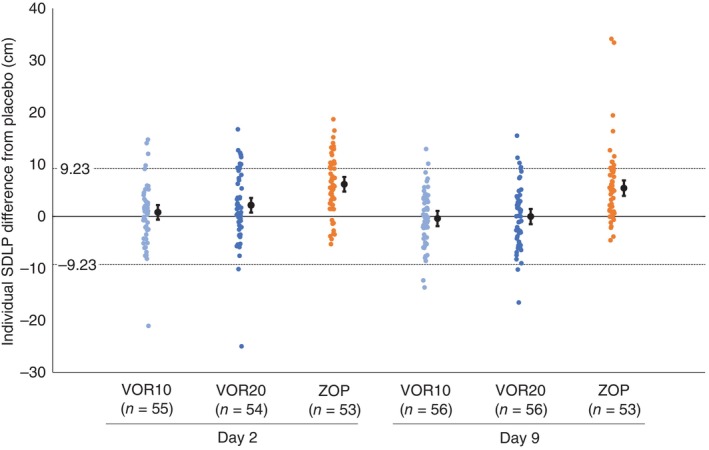
Individual SDLP changes from placebo, mean and 90% confidence interval by treatment group and day. The upper horizontal dotted line indicates clinically significant thresholds (9.23 cm) and the lower line indicates improvement at the same magnitude (−9.23 cm). Individual SDLPs are represented by colored dots. Black filled circles with error bars indicate means for each group with 90% confidence interval. SDLP, standard deviation of lateral position; VOR10, vornorexant 10 mg; VOR20, vornorexant 20 mg; ZOP, zopiclone.

Symmetry analysis of individual ΔSDLP demonstrated that the proportion of participants with ΔSDLP ≥9.23 cm in VOR10 on Days 2 and 9 and in VOR20 on Day 9 were not significantly larger than the proportion of participants with ΔSDLP of ≤ −9.23 cm. In contrast, the distribution of individual ΔSDLP in ZOP showed statistically significant asymmetry on Days 2 and 9 (Fig. 2 and Table 2).

**Table 2:** Symmetry analysis of the proportion of participants whose ΔSDLP exceeded 9.23 cm (indicating impairment) or was below −9.23 cm (indicating improvement).

			≤ −9.23	≥ 9.23		
Group	Day	*n*	*n* (%)	*n* (%)	Test statistic^†^	Reject H_0_ ^‡^
VOR 10 mg	2	55	1 (1.8)	4 (7.3)	1.342	No
	9	56	2 (3.6)	2 (3.6)	0.000	No
VOR 20 mg	2	54	2 (3.7)	11 (20.4)	2.496	Yes
	9	56	2 (3.6)	5 (8.9)	1.134	No
ZOP	2	53	0 (0.0)	17 (32.1)	4.123	Yes
	9	53	0 (0.0)	11 (20.8)	3.317	Yes

VOR, vornorexant; ZOP, zopiclone.

^†^ Generalized sign test.

^‡^ Reject null hypothesis (the proportion of participants categorized in ≤ −9.23 = the proportion of participants categorized in ≥9.23) if test statistic >1.67.

In the *post hoc* analysis, the change in ΔSDLP from Day 2 to Day 9 was calculated for VOR10, VOR20, and ZOP (Table 3). The reduction in SDLP was not statistically significant in any group.

**Table 3:** *Post hoc* analysis result of the change in ΔSDLP from Day 2 to Day 9 in vornorexant 10 and 20 mg and zopiclone groups.

		Change in ΔSDLP (cm)	
Group	*n*	Point estimate	95% CI
VOR 10 mg	55	−0.951	[−2.864, 0.961]
VOR 20 mg	54	−1.688	[−3.623, 0.247]
ZOP	51	−0.904	[−2.694, 0.887]

CI, confidence interval; SDLP, standard deviation of lateral position; VOR, vornorexant; ZOP, zopiclone.

**Fig. 3:**
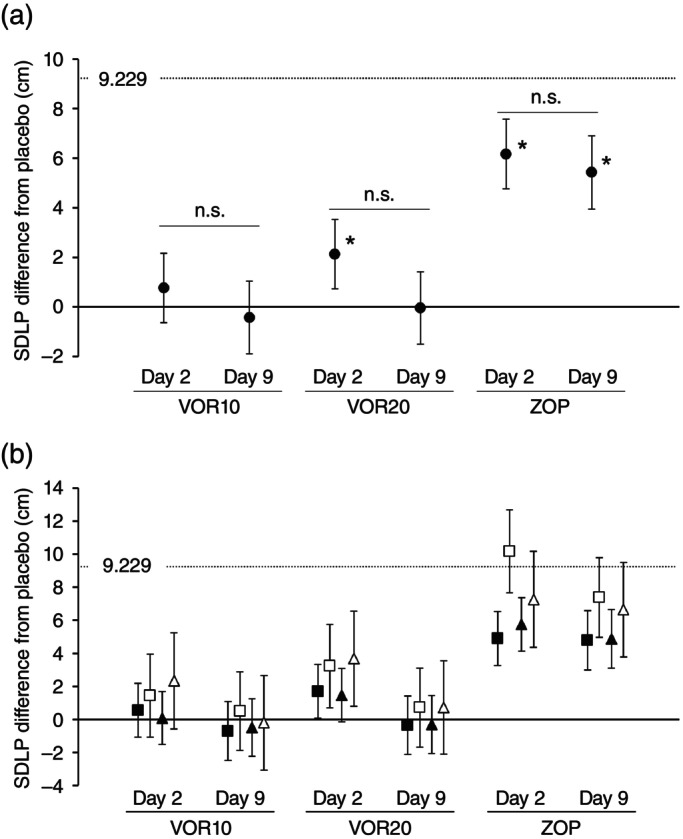
Least square‐mean ΔSDLP by treatment group and day. (a) a visual summary of ΔSDLP in overall population. * the lower bound of 90% confidence intervals exceed 0 cm, indicating statistical significance from placebo. n.s., not significant between Days 2 and 9. (b) subgroup analysis for ΔSDLP by age or sex. Filled square, adults; open square, elderly; filled triangle, male; open triangle, female. Filled or open symbols and error bars indicate point estimates of the mean and 90% confidence intervals. SDLP, standard deviation of lateral position; VOR10, vornorexant 10 mg; VOR20, vornorexant 20 mg; ZOP, zopiclone.

A visual summary of the mean analysis and the *post hoc* analysis in ΔSDLP described above are shown in Fig. 3a.

The subgroup analyses of age and sex are shown in Fig. 3b and Table 4. The ΔSDLP in elderly (age ≥65 years) participants was slightly higher than adult (age <65 years) participants, and the ΔSDLP in female participants was also slightly higher than male participants. It should be noted that 90% CI with male in VOR20 on Day 2 included 0 cm showing non‐significance from PBO, and 90% CI with elderly and female in ZOP on Days 2 and 9 included 9.229 cm showing clinically meaningful impairment.

**Table 4:** Subgroup analysis of SDLP regarding age and sex. SDLP, standard deviation of lateral position.

		SDLP (cm)		ΔSDLP (cm)		
Group	Day	*n*	Mean ± SD	*n*	Point estimate	90% CI
Adult (<65 years)						
PBO	2	42	34.305 ± 7.884	‐	‐	‐
	9	43	34.569 ± 8.180	‐	‐	‐
VOR 10 mg	2	43	34.954 ± 8.004	42	0.554	[−1.071, 2.179]
	9	43	33.823 ± 7.847	43	−0.700	[−2.480, 1.080]
VOR 20 mg	2	44	35.905 ± 8.113	41	1.699	[0.070, 3.328]
	9	44	34.445 ± 8.234	43	−0.353	[−2.126, 1.421]
ZOP	2	44	40.343 ± 9.696	40	4.901	[3.267, 6.536]
	9	43	39.675 ± 10.672	41	4.790	[3.000, 6.579]
Elderly (≥65 years)						
PBO	2	13	40.422 ± 9.914	‐	‐	‐
	9	13	39.348 ± 9.723	‐	‐	‐
VOR 10 mg	2	13	41.913 ± 13.710	13	1.442	[−1.075, 3.959]
	9	13	39.902 ± 11.341	13	0.506	[−1.868, 2.880]
VOR 20 mg	2	13	43.958 ± 10.822	13	3.229	[0.702, 5.757]
	9	13	40.369 ± 9.128	13	0.715	[−1.670, 3.100]
ZOP	2	13	50.739 ± 11.491	13	10.175	[7.659, 12.692]
	9	12	45.893 ± 11.078	12	7.377	[4.959, 9.795]
Male						
PBO	2	39	36.900 ± 8.901	‐	‐	‐
	9	39	36.614 ± 9.356	‐	‐	‐
VOR 10 mg	2	39	36.958 ± 9.713	39	0.082	[−1.521, 1.686]
	9	39	36.094 ± 9.735	39	−0.496	[−2.240, 1.247]
VOR 20 mg	2	39	37.831 ± 9.758	38	1.473	[−0.140, 3.087]
	9	39	36.293 ± 8.819	39	−0.308	[−2.052, 1.436]
ZOP	2	39	42.609 ± 10.876	38	5.751	[4.136, 7.366]
	9	37	41.166 ± 10.823	37	4.874	[3.105, 6.643]
Female						
PBO	2	16	32.950 ± 7.798	‐	‐	‐
	9	17	33.533 ± 6.773	‐	‐	‐
VOR 10 mg	2	17	35.677 ± 10.681	16	2.330	[−0.578, 5.238]
	9	17	33.264 ± 7.082	17	−0.203	[−3.058, 2.651]
VOR 20 mg	2	18	37.547 ± 8.634	16	3.680	[0.801, 6.559]
	9	18	34.721 ± 8.681	17	0.734	[−2.093, 3.561]
ZOP	2	18	42.942 ± 11.416	15	7.263	[4.354, 10.171]
	9	18	40.756 ± 11.575	16	6.637	[3.783, 9.490]

PBO, placebo; SD, standard deviation; VOR, vornorexant; ZOP, zopiclone.

### Secondary endpoints

The mean ILC values of VOR10, VOR20 and ZOP on Day 2 were larger than those of PBO (Table 5). In contrast, only ZOP showed a larger ILC than PBO on Day 9. No notable differences in SDS or TCO were observed between groups. However, individual TCO results showed that the number of participants whose TCO ≥1 was 3 in the ZOP group (1 and 2 on Days 2 and 9, respectively) and 1 in the PBO group on Day 2, whereas a TCO was not recorded for participants in the VOR10 and VOR20 treatment groups.

**Table 5:** Secondary endpoint results in driving test (ILC, SDS, TCO) by administration groups and day.

Endpoint (unit)	Day	*n*	Mean ± SD	*n*	Difference from PBO Mean ± SD
ILC (times)					
PBO	2	55	128.7 ± 86.8	‐	‐
	9	56	138.5 ± 91.3	‐	‐
VOR 10 mg	2	56	144.3 ± 95.4	55	11.9 ± 79.7
	9	56	137.5 ± 96.6	56	−0.9 ± 75.1
VOR 20 mg	2	57	148.8 ± 98.2	54	19.3 ± 87.5
	9	57	138.7 ± 100.1	56	−0.6 ± 73.7
ZOP	2	57	186.1 ± 94.8	53	52.0 ± 67.3
	9	55	180.2 ± 97.7	53	44.8 ± 65.6
SDS (km/h)					
PBO	2	55	4.546 ± 1.379	‐	‐
	9	56	4.469 ± 0.944	‐	‐
VOR 10 mg	2	56	4.726 ± 1.159	55	0.186 ± 0.778
	9	56	4.631 ± 1.174	56	0.162 ± 0.898
VOR 20 mg	2	57	4.641 ± 1.060	54	0.044 ± 0.903
	9	57	4.626 ± 1.264	56	0.157 ± 1.023
ZOP	2	57	4.765 ± 1.126	53	0.204 ± 1.048
	9	55	4.751 ± 1.285	53	0.297 ± 1.096
TCO (times)					
PBO	2	55	0.0 ± 0.3	‐	‐
	9	56	0.0 ± 0.0	‐	‐
VOR 10 mg	2	56	0.0 ± 0.0	55	0.0 ± 0.3
	9	56	0.0 ± 0.0	56	0.0 ± 0.0
VOR 20 mg	2	57	0.0 ± 0.0	54	0.0 ± 0.3
	9	57	0.0 ± 0.0	56	0.0 ± 0.0
ZOP	2	57	0.1 ± 0.4	53	0.0 ± 0.3
	9	55	0.0 ± 0.2	53	0.0 ± 0.2

ILC, inappropriate line crossing; PBO, placebo; SD, standard deviation; SDS, standard deviation of speed; TCO, total course out; VOR, vornorexant; ZOP, zopiclone.

No noticeable trends were observed among all groups in the KSS, DSST, Word recall test, or TMD scores of the POMS 2 (Table S2). A shorter sSL and sWASO and longer sTST were noted in the VOR10, VOR20, and ZOP groups than in the PBO group.

### Safety

The proportion of patients who reported AEs in the PBO, VOR10, VOR20, and ZOP groups were 12.5%, 22.8%, 25.9%, and 20.7%, respectively (Table 6). No deaths or other severe AEs were reported. One participant in the PBO (nasopharyngitis) and one participant in the ZOP group (COVID‐19 infection) experienced AEs leading to discontinuation. The most common AEs reported by ≥5% of the participants in any of the groups were somnolence and dysgeusia. None of the participants reported sickness relating to the simulator. No clinically relevant changes were observed in body weight, laboratory values, vital signs, or standard 12‐lead ECG in any of the groups. No individuals with suicidal ideation or behavior were observed by C‐SSRS assessment throughout the study.

**Table 6:** Summary of adverse events reported by at least 5% of participants in any administration group.

	PBO	VOR 10 mg	VOR 20 mg	ZOP
	*n* = 56	*n* = 57	*n* = 58	*n* = 58
	*n* (%)	*n* (%)	*n* (%)	*n* (%)
Adverse event	7 (12.5)	13 (22.8)	15 (25.9)	12 (20.7)
Somnolence	1 (1.8)	8 (14.0)	12 (20.7)	6 (10.3)
Dysgeusia				5 (8.6)

PBO, placebo; VOR, vornorexant; ZOP, zopiclone.

### Pharmacokinetics

The mean (SD) plasma concentrations of vornorexant at 10.5 h after administration on Days 2 and 9 were 17.4 (14.8) and 15.3 (14.2) ng/mL in VOR10, and 36.9 (29.7) and 33.9 (29.1) ng/mL in VOR20, respectively. No differences were observed between Days 2 and 9 for each dose, and the plasma concentration increased with increasing dose. The mean (SD) serum concentrations of ZOP 10.5 h after administration were 16.96 (6.74) and 16.38 (6.86) ng/mL on Days 2 and 9, respectively. The estimated regression slope (95% CI) between the plasma concentration of vornorexant (ng/mL) and ΔSDLP (cm) was 0.0655 (0.0188, 0.112) and 0.0365 (−0.00582, 0.0788) on Days 2 and 9, respectively.

## Discussion

The primary objective of this study was to assess the next morning residual effects of vornorexant on automobile driving after single and repeated bedtime dosing. Analysis with the upper bounds of 90% CI in ΔSDLP for vornorexant dosing groups falling below 9.229 cm indicated no clinically relevant driving impairments with vornorexant treatment (up to 20 mg) 9 h after dosing. In addition, results with the 90% CI in ΔSDLP for VOR10 on Days 2 and 9 and VOR20 on Day 9 including 0 cm suggested no difference in driving impairment between vornorexant and PBO, except for the single 20 mg dosing. The lack of residual effect of vornorexant is thought to be due to the short half‐life of the drug.

In the ZOP group, the lower bound of 90% CI in ΔSDLP exceeded 0 cm, indicating assay sensitivity. This finding appears consistent with current driving performance assay systems, including both ORT and DS.^14^, ^15^, ^16^, ^17^ In contrast to assay sensitivity, driving studies using ZOP have shown various results on whether ZOP showed a clinically relevant impairment. The upper bound of 90% CI for ZOP did not reach the threshold of 9.229 cm in the present study, which meant a significantly smaller impairment in ZOP; previous reports have shown greater, smaller, or non‐significant impairments in ZOP compared to clinically relevant thresholds.^14^, ^15^, ^16^, ^17^ Additionally, previous studies have demonstrated both statistically higher and comparable odds ratios of accident risk in ZOP users.^23^, ^24^, ^25^ This variability supports the interpretation that the present study was not required to demonstrate clinically significant residual impairment from ZOP as an active control for driving impairment but instead provided sufficient data to confirm positive assay sensitivity.

The primary results demonstrated that neither single nor multiple doses of vornorexant (10 and 20 mg) produced clinically meaningful impairments in driving performance. However, the single 20 mg dose showed a statistically significant increase in SDLP compared to the placebo. These results imply some risks that may be induced by the first administration with high exposure at extratherapeutic level. This is reflected in the result of the symmetry analysis of ΔSDLP in VOR20 single administration. The PK–PD slope was steeper on Day 2, also indicating more pronounced early exposure effects. Variability in drug clearance exists across individuals and therefore caution is required for the first administration of vornorexant, especially in populations that potentially present with unexpectedly high exposure to the drug.

As shown in Fig. 3b and Table 4, females and elderly participants showed numerically higher ΔSDLPs, although not statistically significant in overall population. These findings suggest a potential sensitivity in these subgroups, which should be advised individually. Further pharmacokinetic–pharmacodynamic modeling incorporating sex and age is warranted to explore these observations.

The residual effects in ΔSDLP numerically decreased from Day 2 to Day 9 in the VOR10, VOR20, and ZOP groups, despite no statistical significance in the *post hoc* analysis. Pharmacokinetic factors do not explain this change, as plasma concentrations of vornorexant after single and repeated dosing were similar in a phase 1 study,^12^ and pharmacokinetic analysis in the present study showed a similar trend between Days 2 and 9. The regression analysis of pharmacokinetics, however, supported the reduction in ΔSDLP with smaller estimated regression slope on Day 9 than on Day 2. This transient impairment at a single dose could be attributed to the initial sensitivity to the drug, which was not observed with repeated dosing. Similar adaptation effects have been reported with other orexin receptor antagonists,^14^, ^17^ other hypnotic agents^26^, ^27^ and alcohol,^28^ suggesting that behavioral tolerance may play a role in mitigating next‐morning residual effects over time. Adaption to, or learning from, repeated driving tests should also be considered. Future studies incorporating randomized test sequences or crossover designs with washout periods could help to differentiate between these mechanisms.

Driving outcomes other than the SDLP showed some variation. The ΔILC were increased with incremental doses of vornorexant, and was far larger in ZOP treatment than in vornorexant treatment groups. Furthermore, all ΔILC on Day 9 were smaller than on Day 2, in accordance with changes of ΔSDLP. Previous research has reported that SDS was less sensitive for the detection of drug effects than SDLP.^29^ However, other orexin receptor antagonist has been reported to show statistically significant impairments in SDS, compared to placebo treatment, in parallel with SDLP.^14^ This measurement may be able to detect driving impairment as well, depending on the drug or the driving study method, although the results of SDS in both vornorexant and ZOP treatment groups were not different from the placebo group in this study. No difference in TCO was observed in both vornorexant and ZOP compared to placebo treatment; this was an expected result given that course out means a serious traffic accident and ZOP did not statistically increase a risk of fatal accident in a meta‐analysis of epidemiological studies.^26^ However, the number of participants whose TCO ≥1 was 1 and 2 on Days 2 and 9 in ZOP, respectively, raising the possibility of detecting some signals by other evaluation method with TCO‐specific high assay sensitivity.

It has been reported that cognitive function can influence driving skills.^30^ In this study, no clinically meaningful decrements in cognitive and mood function were observed and support the favorable residual effect profile of vornorexant compared to benzodiazepines.^31^, ^32^ Meanwhile, although VOR20 on Day 2 and ZOP on Days 2 and 9 showed statistically significant changes in SDLP compared to PBO but less clinically relevant effects on driving, drug‐induced cognitive and mood decline in DSST, Word recall test and POMS 2 was not suspected in the present study. This discrepancy may be explained by differences in the sensitivity of these measurements. A previous report has indicated that impairment in DSST was associated with a BAC of 0.8 g/L (0.08%) or more.^33^ The residual effect of vornorexant and ZOP at 9 h post‐administration might be small and undetectable with DSST compared to SDLP.

A limitation of this study could be the inclusion of healthy participants instead of patients with insomnia. However, a previous study has reported that healthy adults were more sensitive to the carryover effects of ZOP than patients with insomnia.^34^ Thus, this study was thought to cover the residual effects on driving performance in patients under drug administration. Nevertheless, generalization to insomnia patients—especially those with comorbidities or polypharmacy—should be made cautiously and future post‐marketing real world evidence studies are warranted to assess residual effects in broader clinical settings.

In conclusion, no clinically significant residual effects on driving performance were observed in healthy participants after a single or repeated administration of vornorexant, where 10 mg was considered the estimated highest therapeutic dose and 20 mg considered as the extratherapeutic dose.

## Disclosure statement

NO is an editorial board member of *Psychiatry and Clinical Neurosciences* and a co‐author of this article. To minimize bias, he was excluded from all editorial decision‐making related to the acceptance of this article for publication. NO has received research support or speaker honoraria from, or served as a consultant to Sumitomo Pharma, Otsuka, Viatris, Eisai, Mochida, Kyowa Pharmaceutical Industry, Nihon Medi‐Physics, Nippon Chemiphar, Medical Review, Nippon Boehringer Ingelheim, SUSMED, and Taisho. KI received personal fees from Eisai, Lundbeck Japan, MSD, Otsuka, Sumitomo Pharma, Takeda, Viatris, and DAIICHI SANKYO outside of the submitted work. YM, DK, YI, and IM are employees of Taisho Pharmaceutical Co., Ltd.

## Author contributions

Conception and design of the study, and acquisition and analysis of data: YM, KI, DK, YI, IM and NO. Drafting the manuscript: YM. Manuscript review: KI, DK, YI, IM and NO.

## Supporting information


**Figure S1.** Study flow diagram.
**Figure S2.** Scatter plots of observations and regression analysis of vornorexant (unchanged form) plasma concentration and ΔSDLP.
**Table S1.** Time schedule for each administration period.
**Table S2.** Other secondary endpoints organized by administration group and day.

**Data S1** CONSORT 2025 checklist.

## Data Availability

The data supporting the findings of this study are not available because of privacy policies.
