# Supplementary material for: Driving performance in the morning after bedtime vornorexant administration: A randomized clinical trial using a driving simulator
Source: Psychiatry Clin Neurosci. 2025 Aug 23;79(11):757–64. doi: 10.1111/pcn.13888 (PMC12580603; doi:10.1111/pcn.13888)
Supplement: Supplementary file 1 — Figure S1. Study flow diagram. Figure S2. Scatter plots of observations and regression analysis of vornorexant (unchanged form) plasma concentration and ΔSDLP. Table S1. Time schedule for each administration period. Table S2. Other secondary endpoints organized by administration group and day. [file PCN-79-757-s001.pdf]

Supporting information

Table S1. Time schedule for each administration period. C-SSRS, Columbia-Suicide Severity Rating Scale; DS, driving simulator; KSS, Karolinska Severity Scale; POMS2, Profile of Mood States 2; DSST, digit symbol substitution test.

	**Time from administration**	
Day1/8		Admission
		Clinical routine tests†, C-SSRS‡
		DS test driving for 5 minutes
		Dinner
	0	Investigational drug administration
	5 min	Going to bed
Day2/9	8 hr	Waking up
		Sleep questionnaire
		Vital sign check, medical examination
		Breakfast
	8.5 hr	KSS, POMS2
	9 - 10 hr	DS driving
	10.5 hr	Pharmacokinetics and laboratory test§
		DSST
	12 hr	Word recall test (Immediate recall)
		DSST (Interference task)
	13 hr	Word recall test (Delayed recall)
		Medical examination, C-SSRS§
		Discharge

†Body weight, laboratory test (only on Day 1), pregnancy test(only on Day 1), vital sign check, 12-lead ECG, and medical examination. ‡Conducted only on Day 1. §Conducted only on Day 9.

Table S2. Other secondary endpoints organized by administration groups and day. PBO, placebo; VOR, vornorexant; ZOP, zopiclone; KSS, Karolinska Sleepiness Scale; DSST, digit symbol substitution test; sSL, subjective sleep latency; sWASO, subjective wake time after sleep onset; sTST, subjective total sleep time; sNAW, subjective number of awakenings; POMS2, Profile of Mood States 2.

Endpoint (unit)	Day	n	Mean ± SD
**KSS**			
PBO	2	55	2.9 ± 1.5
	9	55	3.3 ± 1.7
VOR 10 mg	2	56	3.4 ± 1.7
	9	56	3.3 ± 1.5
VOR 20 mg	2	58	3.8 ± 2.0
	9	57	3.6 ± 1.9
ZOP	2	56	3.2 ± 1.7
	9	57	3.7 ± 1.8
**DSST (number correct)**			
PBO	2	56	61.1 ± 9.5
	9	56	62.5 ± 9.4
VOR 10 mg	2	56	61.7 ± 11.0
	9	56	63.9 ± 9.6
VOR 20 mg	2	58	61.6 ± 9.8
	9	57	65.4 ± 11.1
ZOP	2	58	59.4 ± 10.2
	9	57	62.3 ± 11.6
**Word-recall test, Immediate recall (number correct)**			
PBO	2	56	15.5 ± 3.3
	9	56	15.3 ± 3.3
VOR 10 mg	2	56	15.7 ± 3.4
	9	56	15.7 ± 3.0
VOR 20 mg	2	58	15.7 ± 3.1
	9	57	15.8 ± 3.3
ZOP	2	57	14.9 ± 3.8
	9	57	14.9 ± 3.6
**Word-recall test, Delayed recall (number correct)**			
PBO	2	56	17.2 ± 3.7
	9	56	17.1 ± 3.6
VOR 10 mg	2	56	17.7 ± 3.2
	9	56	17.4 ± 3.1

Endpoint (unit)	Day	n	Mean ± SD
VOR 20 mg	2	58	17.1 ± 3.5
	9	57	17.4 ± 3.5
ZOP	2	57	16.2 ± 4.4
	9	57	16.3 ± 4.0
**Word-recall test, Difference between Immediate recall and Delayed recall (number correct)**			
PBO	2	56	0.7 ± 1.5
	9	56	0.5 ± 1.9
VOR 10 mg	2	56	0.4 ± 1.7
	9	56	0.6 ± 1.9
VOR 20 mg	2	58	1.2 ± 2.3
	9	57	0.5 ± 1.6
ZOP	2	57	1.1 ± 1.9
	9	57	1.0 ± 1.8
**Sleep questionnaire (sSL) (min)**			
PBO	2	56	29.6 ± 34.4
	9	56	27.4 ± 35.0
VOR 10 mg	2	56	20.4 ± 32.6
	9	56	18.3 ± 19.2
VOR 20 mg	2	58	19.2 ± 18.7
	9	57	24.6 ± 35.5
ZOP	2	58	16.8 ± 18.2
	9	57	16.3 ± 17.7
**Sleep questionnaire (sWASO) (min)**			
PBO	2	56	31.6 ± 37.3
	9	56	32.7 ± 39.0
VOR 10 mg	2	56	20.9 ± 30.7
	9	56	30.6 ± 50.1
VOR 20 mg	2	58	20.5 ± 26.9
	9	57	23.8 ± 28.0
ZOP	2	58	15.3 ± 20.4
	9	57	15.1 ± 26.1
**Sleep questionnaire (sTST) (min)**			
PBO	2	56	418.6 ± 52.3
	9	56	419.8 ± 57.4
VOR 10 mg	2	56	438.4 ± 47.1
	9	56	430.9 ± 53.7

Endpoint (unit)	Day	n	Mean ± SD
VOR 20 mg	2	58	439.9 ± 37.1
	9	57	431.3 ± 47.5
ZOP	2	58	447.6 ± 29.4
	9	57	448.4 ± 34.9
**Sleep questionnaire (sNAW)**			
PBO	2	56	2.3 ± 2.3
	9	56	2.1 ± 2.0
VOR 10 mg	2	56	2.0 ± 1.5
	9	56	2.0 ± 1.9
VOR 20 mg	2	58	1.9 ± 1.6
	9	57	2.1 ± 1.5
ZOP	2	58	1.6 ± 1.9
	9	57	1.5 ± 1.9
**POMS2 (Total mood disturbance score)**			
PBO	2	55	40.2 ± 5.7
	9	55	40.8 ± 6.9
VOR 10 mg	2	56	39.9 ± 5.1
	9	56	39.3 ± 5.0
VOR 20 mg	2	58	40.7 ± 6.3
	9	57	40.6 ± 5.7
ZOP	2	57	39.7 ± 4.5
	9	57	40.6 ± 5.2

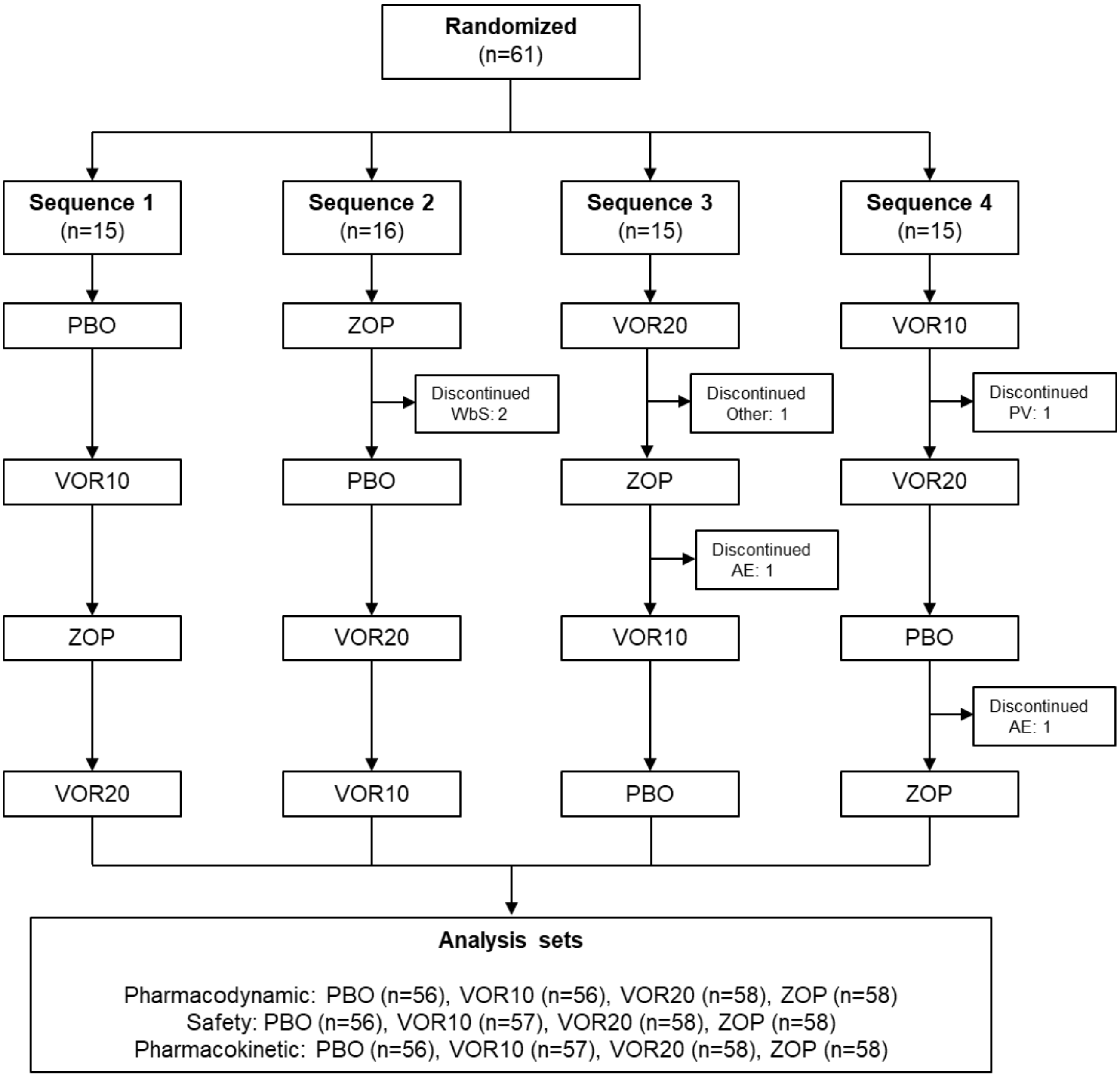

Figure S1. Study flow diagram. AE, adverse event; PBO, placebo; PV, protocol violation; VOR, vornorexant; WbS, withdrawal by subject; ZOP, zopiclone.

a

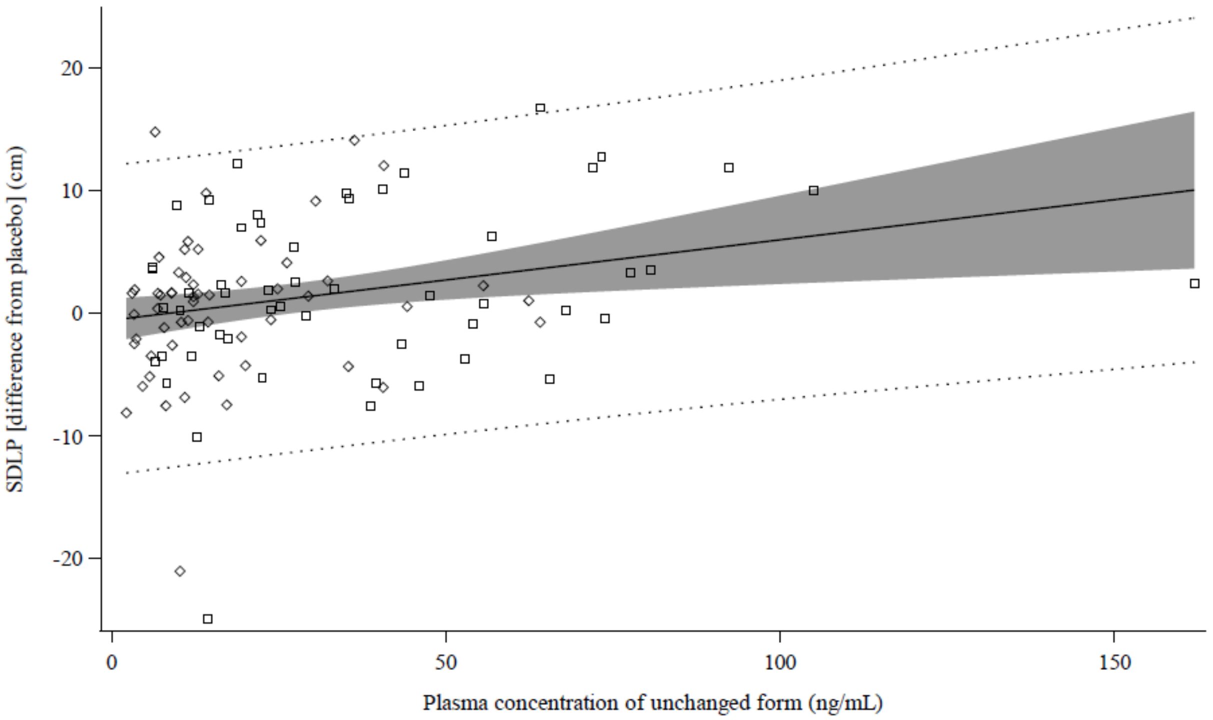

b

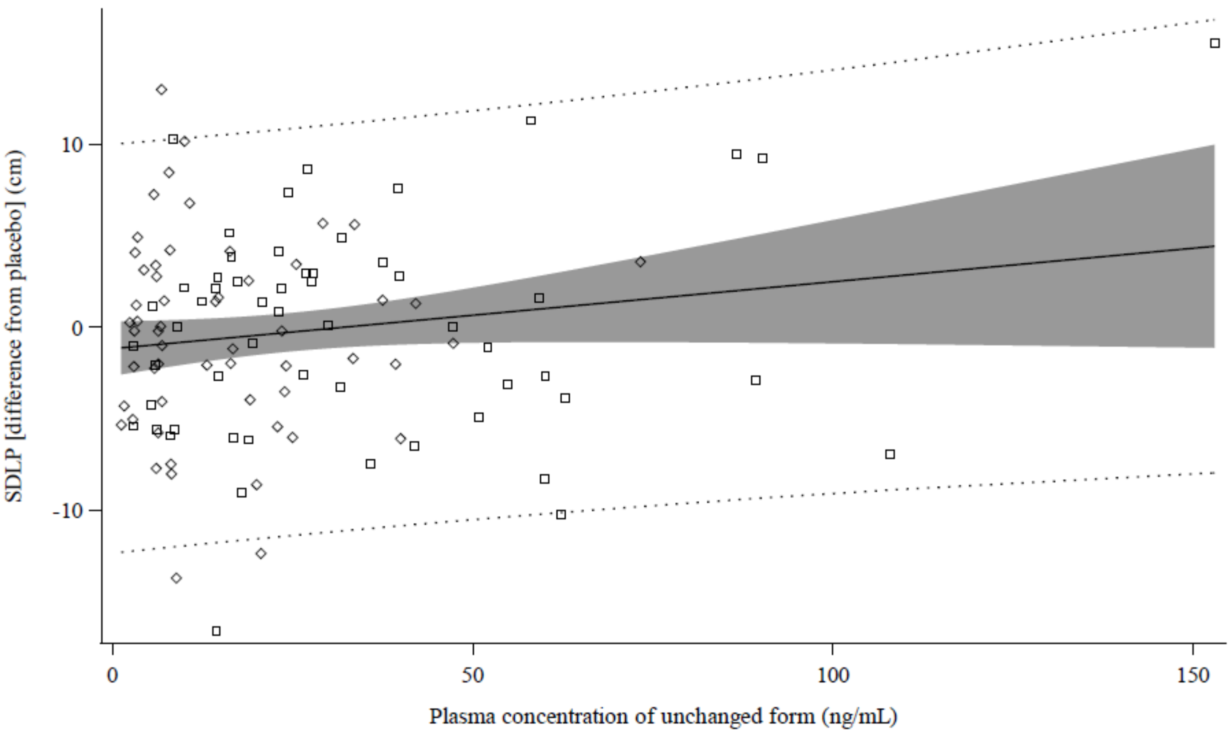

Figure S2. Scatter plots of observations and regression analysis of vornorexant (unchanged form) plasma concentrations and ΔSDLP. a: Day 2, b: Day 9. Solid line, regression line; gray area, 95% confidence interval of the regression line; dotted line, 95% prediction limits (upper and lower);

diamond marks, vornorexant 10 mg; square marks, vornorexant 20 mg. SDLP, standard deviation of lateral position.
